# Next Generation Sequencing Based Non-invasive Prenatal Testing (NIPT): First Report From Saudi Arabia

**DOI:** 10.3389/fgene.2021.630787

**Published:** 2021-02-04

**Authors:** Yusra Alyafee, Abeer Al Tuwaijri, Qamre Alam, Muhammad Umair, Shahad Haddad, Mashael Alharbi, Maryam Ballow, Mohammed Al Drees, Abdulkareem AlAbdulrahman, Aziza Al Khaldi, Majid Alfadhel

**Affiliations:** ^1^Medical Genomics Research Department, King Abdullah International Medical Research Center (KAIMRC), King Saud Bin Abdulaziz University for Health Sciences, King Abdul Aziz Medical City, Ministry of National Guard Health Affairs, Riyadh, Saudi Arabia; ^2^Department of Pathology and Laboratory Medicine, King Abdulaziz Medical City, Ministry of National Guard Health Affairs (MNG-HA), Riyadh, Saudi Arabia; ^3^Genetics and Precision Medicine Department (GPM), King Abdullah Specialized Children’s Hospital, King Saud Bin Abdulaziz University for Health Sciences, King Abdulaziz Medical City, MNG-HA, Riyadh, Saudi Arabia

**Keywords:** aneuploidy, chromosomal duplications, deletions, fetal DNA, next generation sequencing, non-invasive prenatal testing (NIPT)

## Abstract

**Background:** Non-invasive prenatal testing (NIPT) for aneuploidy in pregnant women screening has been recently established in Saudi Arabia. We aim from this study to report our experience in the implementation of this new technology in clinical practice and to assess factors influencing cell-free fetal (cffDNA) fraction and successful NIPT reporting.

**Methods:** In total, 200 pregnant women were subjected to the NIPT test using standard methods. Next-generation sequencing (NGS) was used to analyze cffDNA in maternal plasma.

**Results:** Out of the 200 NIPT cases, the average age of pregnant women was 35 ± 6 years (range: 21–48 years). The average cffDNA fraction of reported cases was 13.72% (range: 3–31%). Out of these 200 cases, 187 (93.5%) were at low risk, while 13 (6.5%) cases revealed high risk for aneuploidy. Among these chromosomal abnormalities, 7 (3.5%) cases of Down’s syndrome, 5 (2.5%) Edwards’ Syndrome, and only 1 case of (0.5%) Patau’s syndrome was observed. Out of the 13 high-risk cases, 2 (15.3%) were found in women below the age of 30.

**Conclusion:** This is the first study reporting the successful implementation of an in-house NIPT screening service in Saudi Arabia. Our data showed high accuracy and sensitivity to detect high-risk cases indicating the usefulness of such a technique as an alternative to invasive testing and (hopefully) will change the common screening practice for pregnant women in Saudi Arabia.

## Introduction

Congenital abnormalities in the fetus are considered one of the most important causes of infant death (Wapner and Lewis, 2002). One type of congenital abnormality is aneuploidy, which is defined as the gain or loss of one or more chromosomes from the normal chromosome number (Avent et al., 2009). Down syndrome or trisomy 21 (T21) is one of the most common chromosomal abnormalities, occurring with a frequency of 1 per 800 live births (Ehrich et al., 2011). Trisomy 18 (T18) Edward syndrome and trisomy 13 (T13) or Patau syndrome are also among the high prevalent autosomal aneuploidy with an incidence of (1 per 5,000 live births for Trisomy 13 and 1 per 16,000 live births for Trisomy 13, respectively) (Baty et al., 1994; Hassold and Hunt, 2001). In Saudi Arabia, 6.7% of recurrent pregnancy loss is caused by chromosomal abnormalities (Al-Ghamdi and Makhashen, 2016), and Down syndrome is considered the most common chromosomal anomaly with a prevalence of 6.6 per 10,000 children (Al Salloum et al., 2015).

In 1997, Lo et al. (1997) reported that cffDNA can be quantified in the plasma from pregnant women. Subsequently, this finding paved the way for developing new applications in clinical practice that relied on analyzing this fetal genetic material, i.e., detecting fetal sex and Rh blood group type (Rather et al., 2019). cffDNA comprises of approximately 3–13% of the mother’s cell-free DNA and is released into maternal circulation from placental cells undergoing apoptosis. The amount of cffDNA increases with increasing gestational age and is cleared hours after delivery (American College of Obstetricians and Gynecologists (ACOG), 2016). The identification of cffDNA in the maternal circulation accompanying rapid advances in sequencing technology has allowed the analysis of circulating cffDNA to be executed with considerable sensitivity and specificity. Since 2011, Non-invasive prenatal testing (NIPT) has been recommended as an extremely accurate method for pregnant women with a high risk of fetal aneuploidy by the American College of Obstetricians and Gynecologists and the Society for Maternal-Fetal Medicine (American College of Obstetricians and Gynecologists (ACOG), 2015).

Currently, in most practices, prenatal diagnosis utilizes non-invasive methods to screen for fetal anomalies. High-risk results will be followed by invasive tests such as amniocentesis and transcervical Chronic Villus Sampling (CVS), which may increase the risk of spontaneous abortion of 1 in 455 and 1 in 900 pregnancies, respectively (Carlson and Vora, 2017).

Validation studies showed that the NIPT sensitivity for detecting T21 Downs Syndrome was nearly 99%, whereas the sensitivity for detecting T18 Edwards Syndrome and T13 Patau Syndrome was 88–100%, and the specificity was 100% (Ashoor et al., 2012; Sparks et al., 2012a; Zimmermann et al., 2012). The overall reported detection rate for T13, T18, and T21 was 98.9% with a false positive rate of 1.4% (Devers et al., 2013).

Currently, NIPT is not considered a diagnostic tool following recommendations of the American College of Medical Genetics and Genomics (ACMG) and National Society of Genetic Counselors (NSGC) (Devers et al., 2013; Gregg et al., 2016; National Society of Genetic Counselors (NSGC), 2016). As per the ACMG, NIPT can replace conventional screening for Patau, Edwards, and Down syndrome beginning at 9–10 weeks of gestational age and across the maternal age spectrum, and for patients who are not significantly obese (Gregg et al., 2016). In addition, the International Society for Prenatal Diagnosis (ISPD) supports NIPT as a primary test offered to all pregnant women whereas women with high-risk pregnancies should be offered invasive prenatal diagnosis (Benn et al., 2015).

In this present study, we describe our experience in introducing NIPT service in King Abdulaziz Medical City located in Riyadh, Saudi Arabia. Our main goal was to report the implementation of this new technology in clinical practice and change the existing workflow of high-risk pregnancy for aneuploidies. Our second goal was to assess clinically significant factors influencing cffDNA fraction and successful NIPT reporting. In conclusion, to the best of our knowledge, this is the first study highlighting the experience of the integration of NIPT in the medical care setting in Saudi Arabia.

## Materials and Methods

### Study Approval and Consent

This study was approved by the Institutional Review Board (IRB) of King Abdullah International Medical Research Center (KAIMRC), Riyadh, Saudi Arabia (RC19/115/R-Approved July, 2019). The participants went through a full clinical assessment for the genetic and rare diseases at the Obstetrics and Gynecology Clinic of the Ministry of National Guard Hospital (MNGH), Riyadh, Saudi Arabia. A detailed review of the informed consent form was performed with participants, this included details of anonymization and storage of clinical data and dissemination of findings. In addition, informed consent forms were signed by all participants.

### Study Subjects

A total of 200 pregnant women aged between 21 and 43 years were recruited in the current study from October 2019 to August 2020. All participants conceived naturally and had a singleton pregnancy with a gestational age of at least more than 10 weeks. Full and detailed family history for any chromosomal abnormality are obtained together with weight and High for each recruited patient. To confirm the number of fetuses and gestational age, an ultrasound scan was performed on every participant included in this study.

### Blood Samples and Genomic DNA Extraction

Approximately 5–10 ml of the peripheral venous blood sample was collected from each participant for NIPT in a cell-free DNA blood collection EDTA tube (Streck, Omaha, NE, United States) and each tube was allocated an anonymous unique medical record number and accession number. Within 6 h of collection, the blood samples were centrifuged in two steps: first spin at 1,600 rpm for 10 min. Plasma was transferred to 2 ml microcentrifuge tubes followed by the second spin at 14,500 rpm for 12 min to remove remaining residual cells in the samples and kept at −20°C for DNA extraction (Lau et al., 2013). From each plasma samples (2 ml), genomic DNA extraction experiments were performed by using QIAamp Circulating Nucleic Acid Kit from Qiagen (Hilden, Germany) according to the manufacturer’s protocol. The extracted cell-free DNA, which comprises both maternal and fetal cell-free DNA fragments, thus subsequently used for the preparation of DNA libraries (Srinivasan et al., 2013).

### DNA Library Preparation

DNA library preparation was performed using the IONA^®^ Library Preparation Plate-1 kit (United Kingdom). Library preparation consists of the sequential stages namely DNA end repair, barcode adaptor ligation, and PCR amplification. The end repair reaction involves incubation of the DNA sample with an enzyme mix (T4 DNA polymerase, Klenow DNA polymerase, and T4 polynucleotide kinase) to produce blunt-ended DNA fragments. The fragments are also phosphorylated at the 5’ end to allow for subsequent ligation of the adaptor. The thermal cycler condition for end repair reaction was 25°C for 20 min followed by 70°C for 10 min (Sparks et al., 2012c). After the completion of the end repair reaction, the barcode adaptor ligation was performed by using a unique barcode for each sample and enzyme mix (DNA ligase and DNA polymerase) according to the manufacturer protocol. The samples were put in the thermal cycler at 25°C for15 min followed by 65°C for 5 min. Thereafter, an adaptor ligation clean-up reaction was performed before PCR amplification to remove unused adaptors by using IONA^®^ Library Preparation Kit plate-1 9 (United Kingdom) (Sehnert et al., 2011). After the clean-up stage, the PCR amplification reaction was performed by using the PCR mix, which contains a high-fidelity DNA polymerase enzyme and PCR primers designed to bind sequences in the adaptor oligonucleotides, which in turn is used to generate the amplified DNA library. The PCR cycling conditioned was 98°C for 30 s (1 Cycle) followed by 98°C for 10 s, 58°C for 30 s, and 72°C for 30 s (11 cycle) and finally holding at 72°C for 5 min. Moreover, a DNA library multiplex was cleaned up using the paramagnetic bead reagents in the IONA^®^ Library Preparation Kit plate-1 (United Kingdom) to remove unused PCR primers and master mix (Palomaki et al., 2011).

### DNA Library Size Selection and NGS

The DNA libraries were selected according to size so that the fragments lie within the suitable read length range for the NGS platform. First of all, the paramagnetic beads were added to the DNA library pool, which binds to larger unwanted fragments present in the sample, while the supernatant which contains the desired DNA library was retained. Furthermore, the size selected DNA library pool was quantified according to manufacturer protocol (High Sensitivity DNA Reagents & Agilent 2100 Bioanalyzer) (Liao et al., 2014). Thereafter, the DNA library pool and run control provided in the IONA^®^ Library Preparation Kit was diluted to the required concentration for the NGS reaction. Finally, NSG of the multiplexed DNA libraries were performed according to the protocol provided by the manufacturer (Ion Chef and IonS5 XL, Life Technologies, SD, United States), and 12 samples per chip (Ion 540^TM^ Chip-Life Technologies) were analyzed (Sparks et al., 2012b).

### Bioinformatics Analysis of NGS Data

The resulting data were processed by the IONA^®^ Software (Variant Caller, Life Technologies) in comparison with the reference sequence (GRCh37 Sequences), which calculates the likelihood of an affected or unaffected pregnancy for each of the trisomy’s investigated by the IONA^®^ test. The types of fetal chromosome abnormality were predicted through the dynamic threshold method and the quadratic element segmentation algorithm (Lau et al., 2014). All data collected and included were consented and approved.

### Statistical Analysis

After data collection, retrospective statistical analysis was performed by IBM SPSS STATISTICS 26.0. Descriptive data analysis was presented by mean values and standard deviations, while categorized data were compared by percentage. Correlation analysis were also used, and the Pearson value was calculated (significant if ≤ 0.01).

## Results

### Participant Clinical Description

In our study, a total of 200 pregnant women were recruited between October 2019 and August 2020. Almost all the eligible participants were of Arab ethnicity and Saudi nationality, had a single fetus and more than 10 weeks of gestation. The average age of pregnant women was (35.7 ± 6) years with a range between 21 and 48 years. Despite our recommendation to the obstetricians to enroll pregnant women within (10–14) weeks of gestation, the average gestational age at the time of NIPT in this study was 19.14 weeks with a range of 10–32 weeks. As shown in the chart below almost half of the cases 98/200 (49%) underwent NIPT at 10–16 weeks, while 102/200 (51%) at more than 20 weeks. The median body mass index BMI was 30.84 kg/m^2^ and in the range of 15–48 kg/m^2^. Furthermore, of the 200 cases in this study, only 34 (17%) participants had a previous family history for any chromosomal aneuploidy. Detailed clinical descriptions of all participants are summarized in Table 1 below.

**TABLE 1:** Descriptive statistic of 200 pregnant women recruited in this study.

Features	Number	Percentage (%)
**Ethnicity**		
Saudi	199	99.5
Other than Saudi	1	0.5
**Maternal age (year)**		
21–25	15	7.5
26–30	25	12.5
31–35	42	21
36–40	77	38.5
More than 40	41	20.5
Average age	35.69	−
Range	21–48	−
**Gestational age at the time of NIPT (weeks)**		
10–16	98	49
17–20	23	11.5
More than 20	79	39.5
Average gestational age	19.14	−
Range	10–32	−
**Fetal DNA fraction (%)**		
4–10%	60	30
11–20%	123	61.5
More than 20%	17	8.5
Average fetal DNA fraction	13.38	−
Range	4–31%	−
**Gender**		
Male	106	53
Female	93	46.5
Sex determination failure	1	0.5
**Previous history**		
Yes	34	17
No	166	83
**Body mass index (kg/m^2^)**		
Under 18.5 kg/m^2^	3	1.5
Normal weight (18.5–25)	37	18.5
Overweight (25–30)	55	27.5
Obese (over 30)	105	52.5
Average	30.84	−
Range	15–48	−

### Fetal Fraction Outcome and Related Factors

**FIGURE 1:**
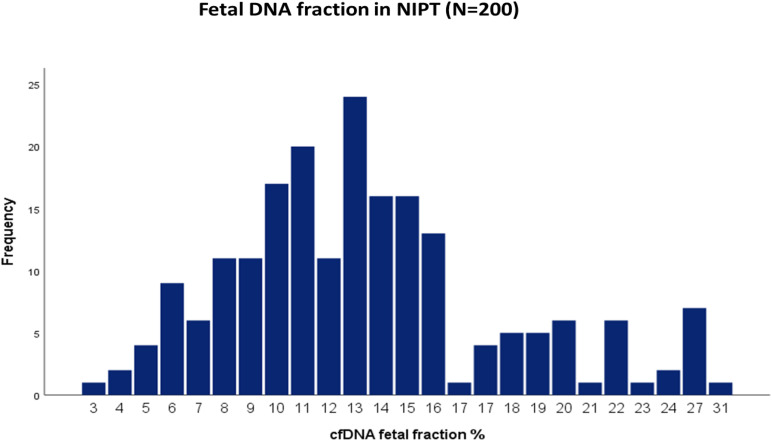
Distribution of cfDNA fetal fraction percentage of all the 200 NIPT cases.

**FIGURE 2:**
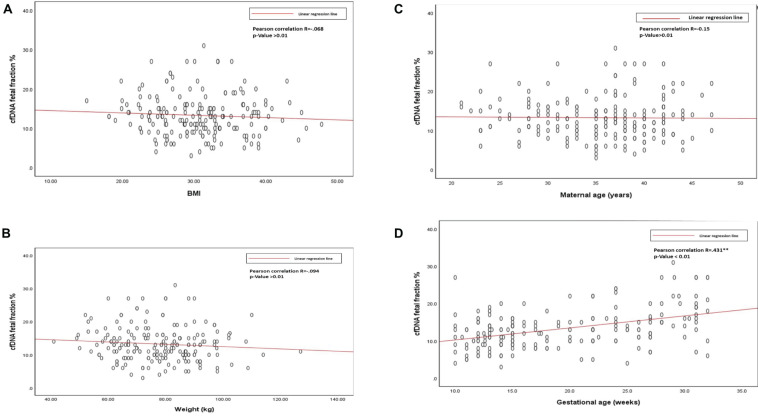
**(A)** Scatterplot of BMI and cfDNA fetal fraction. **(B)** Scatterplot of body weight and cfDNA fetal fraction. **(C)** Scatterplot of maternal age, and cfDNA fetal fraction. **(D)** Scatterplot of gestational age and cfDNA fetal fraction.

The average cffDNA fraction of reported cases was 13.26% and in the range of 4–31%. Among them, 60 cases showed (4–10%) fetal fraction, 123 cases showed (11–20%) while 17 cases obtained a fetal fraction of (≥ 20%) as presented in Figure 1. On the other hand, gestational age showed a positive correlation related to fetal fraction, while maternal age, body weight, and BMI showed no significant correlation to fetal fraction as shown in Figure 2.

### NIPT Screening for Trisomy 21,18,13, and Fetal Gender

Out of 200 cases, 187 (93.5%) were low risk for aneuploidies (Euploidy), while 13 (6.5%) cases were revealed as high risk. Among these chromosomal abnormalities, 7 (3.5%) cases were Down’s syndrome, 5 (2.5%) Edwards’ Syndrome, and only 1 (0.5%) Pat au’s syndrome. As described in Table 3, among these 13 high risk cases 2 (1%) were reported in pregnant women within the age of (21–30 years), an additional 3 cases (1.5%) were reported within the ages of (30–35 years), another 6 cases (3%) within the ages of (36–40 years), and the last 2 cases (1%) in pregnant women ≥ 40 years. Moreover, the results revealed a total of 199 cases of which 103 (53%) of the fetuses were males and 93 (46.5%) were females with only one case of failed sex determination.

### NIPT Performance for Detecting Fetal Trisomy

All the NIPT results obtained were verified by following up the results of invasive prenatal diagnosis through fluorescence *in situ* hybridization (FISH) for high-risk cases and newborn follow-up for low risk cases. All low-risk cases were confirmed to be true negative, while for high-risk cases 7 out 7 (100%) cases of T21, 4 out of 5 (80%) cases of T81, and 1 out of 1 (100%) cases of T13 were found to be true positive. However, we found only one false positive case of T18, no other cases were false negatives. Detailed descriptions of all high-risk cases are summarized in Table 2 below.

**TABLE 2:** Clinical finding of 200 pregnant women recruited for NIPT.

Chromosome aneuploidy	Number	Percentage (%)
Total	200	100
Low risk	187	93.5
High risk (T21)	7	3.5
High risk (T18)	5	2.5
High risk (T13)	1	0.5
Total high risk aneuploidy (T21,T18, and T13)	13	6.5
False positive for T21	Nil	−
False positive for T18	1	0.5
False positive for T13	Nil	−
**Total false positive (T21,T18, and T13)**	**1**	**0.5**
**Total false negative (T21,T18, and T13)**	**Nil**	−

*Bold values indicates that total number of the cases.*

## Discussion

Fetal chromosomal abnormalities are major reasons for developmental delay and intellectual disability (Capalbo et al., 2017). Golden standards for the detection of chromosomal abnormalities are conventional prenatal testing techniques such as karyotyping, CGH, and microarray-based technologies. These conventional screening methods require testing of fetal fluid through invasive approaches, like amniocentesis, which increases the risk of miscarriage, vaginal bleeding, and intrauterine infection in pregnant women (Cuckle and Maymon, 2016; Pös et al., 2019).

With the discovery of cffDNA by Lo et al. (1997) in pregnant women, NIPT using a high-throughput sequencing method is widely adopted for clinical detection of chromosomal abnormalities (Lo et al., 1997). This high-throughput DNA sequencing technique can effectively detect large-scale genetic mutations in a short time, with high accuracy and specificity for trisomy T21, T18, and T13. Since 2011, NIPT has been recommended by the American College of Obstetricians and Gynecologists and the Society for Maternal-Fetal Medicine as an extremely accurate method of the detection of fetal aneuploidy for high risk pregnant women (Gregg et al., 2016).

**FIGURE 3:**
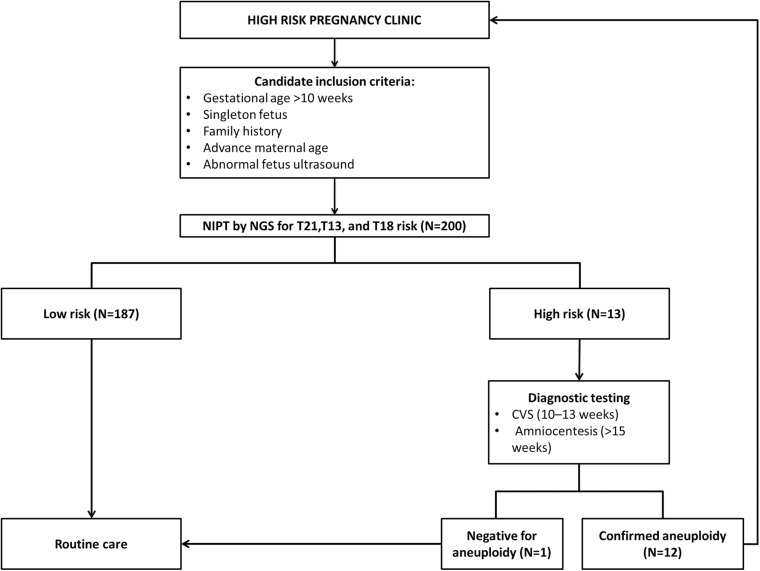
Study summary for all the NIPT recruited cases.

Out of the total number of participants, 13 (6.5%) of them showed chromosomal abnormalities (Figure 3). It is well established that maternal age is associated with a rapid decline in the production of healthy and high-quality oocytes (REFERENCE) resulting in reduced fertility in women older than 35 years of age. Maternal age is a major concern for aneuploidy and genetic disorders in the offspring in the context of an increased proportion of mothers having children at older ages (Kuliev et al., 2003). The association of mother’s age and chromosomal aneuploidy in a fetus is also reflected in this study. Among 200 samples investigated in this study, 13 (6.5%) were high-risk cases, out of these 13 cases, 8 (61.5%) were found to be in the pregnant women whose age was more than 36 years. However, we did identify two cases of aneuploidy in the age group 21–30 years. Although previously ACOG Guidelines recommended the use of NIPT in pregnant women age 35 years and older, we found that the age of our high-risk group started earlier which agreed with more recent recommendations of ACOG guidelines to offer NIPT for pregnant women regardless of their age and risk factors (Rose et al., 2020). These clinical findings have been summarized in Table 3. Moreover, high-risk cases were confirmed by an invasive test like amniocentesis for further confirmation of chromosomal abnormalities. However, we only found one “false positive” case which can be attributed to placental mosaicism (Li et al., 2020), no other cases were “false negative.” Hence, NIPT substantially reduced the number of invasive tests (Noh et al., 2019). Additionally, our data showed no correlation between increased BMI of pregnant women and reduced cffDNA fetal fraction, which can be explained by increased gestational age for those women (Livergood et al., 2017). This finding coincides with previously reported results by Flöck et al. (2017).

**TABLE 3:** Correlation of age with high risk for T21, T18, and T13.

Maternal age (Year)	Low risk	High risk for T21, T18, and T13
21–25	13(6.5%)	1(0.5%)
26–30	30(15%)	1(0.5%)
31–35	41(20.5%)	3(1.5%)
36–40	64(32%)	6(3%)
More than 40	39(19.5%)	2 (1%)
Total	**187 (93.5%)**	**13 (6.5%)**

*The bold values is to highlight total number of the cases.*

In summary, this study highlights a successful experience of the implementation of NIPT in the Arab region. Moreover, the central role of such a screening test comes from its ability to detect high risk fetal aneuploidy of chromosome 21,13, and 18 even before any minor or major markers appear in first trimester ultrasound screening. Overall, these findings shed light on the clinical value of NIPT for early detection and intervention in Saudi Arabia, particularly in an era of an increased childbearing age of Saudi women.

## Conclusion

In conclusion, prenatal screening using high throughput DNA sequencing based NIPT is a predominantly secondary screening test in the Kingdom of Saudi Arabia. We have shown that the performance of NIPT can detect Down, Edward, and Patau syndromes with high accuracy and specificity. Therefore, this study offers further evidence of its accuracy, specificity, simplicity, and that it can be performed at an early gestational age. Furthermore, NIPT utilizing a low coverage (0.1×) whole-genome sequencing approach provides a unique possibility to screen for a wider spectrum of fetal chromosomal abnormalities beyond common aneuploidies at an affordable cost. However, it is important to note that one of NIPT’s limitations is that it can produce false positive or false negative results. Therefore, NIPT cannot be considered a confirmatory test, hence, high-risk positive results require further invasive testing for confirmation.

## Data Availability Statement

The original contributions presented in the study are publicly available. This data can be found here: https://databases.lovd.nl/shared/phenotypes/0000245127.

## Ethics Statement

The studies involving human participants were reviewed and approved by King Abdullah International Medical Research Centre, Riyadh, Saudi Arabia (Reference #H-01-R-005) and were performed in accordance with the ethical standards. Written informed consent was obtained. The patients/participants provided their written informed consent to participate in this study.

## Author Contributions

MAF: designing and writing the project. YA: project management, writing, and data analysis. QA: performing the experiment and writing the manuscript. MU and AAT: manuscript writing and editing. SH, MA, MB, MAD, and AAA: performing the experiment. AAK: logistic support. All authors contributed to the article and approved the submitted version.

## Conflict of Interest

The authors declare that the research was conducted in the absence of any commercial or financial relationships that could be construed as a potential conflict of interest.

## Abbreviations

NIPT: Non-Invasive Prenatal Testing
CFF DNA: Cell-Free Fetal DNA
NSG: Next-Generation sequencing
CGH: Comparative Genomic Hybridization
IRB: Institution Review Board
NGH: National Guard Hospital
KAIMRC: King Abdullah International Medical Research Centre
FISH: Fluorescence *In Situ* Hybridization.

## References

[B1] American College of Obstetricians and Gynecologists (ACOG) (2015). Committee opinion no. 640: cell-free DNA screening for fetal aneuploidy. *Obstet. Gynecol.* 126 e31–e37. 10.1097/01.AOG.0000471172.63927.b6 26287791

[B2] American College of Obstetricians and Gynecologists (ACOG) (2016). Practice bulletin no. 163: screening for fetal aneuploidy. *Obstet. Gynecol.* 127 e123–e137. 10.1097/AOG.0000000000001406 26938574

[B3] Al Salloum A. El Mouzan M. I. Al Herbish A. Al Omer A. Qurashi M. (2015). Prevalence of selected congenital anomalies in Saudi children: a community-based study. *Ann. Saudi Med.* 35 107–110. 10.5144/0256-4947.2015.107 26336015 PMC6074139

[B4] Al-Ghamdi A. A. Makhashen S. F. (2016). Etiology of recurrent pregnancy loss in Saudi Females. *Saudi J. Med. Med. Sci.* 4 187–191. 10.4103/1658-631X.188258 30787727 PMC6298350

[B5] Ashoor G. Syngelaki A. Wagner M. Birdir C. Nicolaides K. H. (2012). Chromosome-selective sequencing of maternal plasma cell–free DNA for first-trimester detection of trisomy 21 and trisomy 18. *Am. J. Obstet. Gynecol.* 206:322.e1-5. 10.1016/j.ajog.2012.01.029 22464073

[B6] Avent N. D. Madgett T. E. Maddocks D. G. Soothill P. W. (2009). Cell-free fetal DNA in the maternal serum and plasma: current and evolving applications. *Curr. Opin. Obstet. Gynecol.* 21 175–179. 10.1097/GCO.0b013e3283294798 19262379

[B7] Baty B. J. Jorde L. B. Blackburn B. L. Carey J. C. (1994). Natural history of trisomy 18 and trisomy 13: II. psychomotor development. *Am. J. Med. Genet.* 49 189–194. 10.1002/ajmg.1320490205 7509567

[B8] Benn P. Borrell A. Chiu R. W. Cuckle H. Dugoff L. Faas B. (2015). Position statement from the chromosome abnormality screening committee on behalf of the board of the international society for prenatal diagnosis. *Prenatal Diag.* 35 725–734. 10.1002/pd.4608 25970088

[B9] Capalbo A. Rienzi L. Ubaldi F. M. (2017). Diagnosis and clinical management of duplications and deletions. *Fertil. Steril.* 107 12–18. 10.1016/j.fertnstert.2016.11.002 28040093

[B10] Carlson L. M. Vora N. L. (2017). Prenatal Diagnosis Screening and Diagnostic Tools. *Obstet. Gynecol. Clin. North Am.* 44 245–255. 10.1016/j.ogc.2017.02.004 28499534 PMC5548328

[B11] Cuckle H. Maymon R. (2016). Development of prenatal screening–A historical overview. *Sem. Perinatol.* 40 12–22. 10.1053/j.semperi.2015.11.003 26764253

[B12] Devers P. L. Cronister A. Ormond K. E. Facio F. Brasington C. K. Flodman P. (2013). Noninvasive prenatal testing/noninvasive prenatal diagnosis: the position of the national society of genetic counselors. *J. Genet. Counsel.* 22 291–295. 10.1007/s10897-012-9564-0 23334531

[B13] Ehrich M. Deciu C. Zwiefelhofer T. Tynan J. A. Cagasan L. Tim R. (2011). Noninvasive detection of fetal trisomy 21 by sequencing of DNA in maternal blood: a study in a clinical setting. *Am. J. Obstet. Gynecol.* 204: 205.e1-11. 10.1016/j.ajog.2010.12.060 21310373

[B14] Flöck A. Tu N. C. Rüland A. Holzgreve W. Gembruch U. Geipel A. (2017). Non-invasive prenatal testing (NIPT): Europe’s first multicenter post-market clinical follow-up study validating the quality in clinical routine. *Arch. Gynecol. Obstet.* 296 923–928. 10.1007/s00404-017-4517-3 28887622

[B15] Gregg A. R. Skotko B. G. Benkendorf J. L. Monaghan K. G. Bajaj K. Best R. G. (2016). Noninvasive prenatal screening for fetal aneuploidy, update: a position statement of the American College of Medical genetics and genomics. *Genet. Med.* 18 1056–1065. 10.1038/gim.2016.97 27467454

[B16] Hassold T. Hunt P. (2001). To err (meiotically) is human: the genesis of human aneuploidy. *Nat. Rev. Genet.* 2 280–291. 10.1038/35066065 11283700

[B17] Kuliev A. Cieslak J. Ilkevitch Y. Verlinsky Y. (2003). Chromosomal abnormalities in a series of 6,733 human oocytes in preimplantation diagnosis for age-related aneuploidies. *Reproduct. Biomed. Online* 6 54–59. 10.1016/S1472-6483(10)62055-X 12626143

[B18] Lau T. K. Cheung S. W. Lo P. S. Pursley A. N. Chan M. K. Jiang F. (2014). Non-invasive prenatal testing for fetal chromosomal abnormalities by low-coverage whole-genome sequencing of maternal plasma DNA: review of consecutive cases in a single center. *Ultrasound Obstet. Gynecol.* 43 254–264. 10.1002/uog.13277 24339153

[B19] Lau T. K. Jiang F. Chan M. K. Zhang H. Lo P. S. Wang W. (2013). Non-invasive prenatal screening of fetal down syndrome by maternal plasma DNA sequencing in twin pregnancies. *J. Maternal-fetal Neonatal Med.* 26 434–437. 10.3109/14767058.2012.733768 23035860

[B20] Li J. Xie M. Wang F. Ma J. Li J. Chen C. (2020). A rare case of NIPT discrepancy caused by the placental mosaicism of three different karyotypes, 47,XXX, 47,XX,+21, and 48,XXX,+21. *Mol. Genet. Genom. Med.* 8:e1279. 10.1002/mgg3.1279 32463164 PMC7434741

[B21] Liao C. Yin A. H. Peng C. F. Fu F. Yang J. X. Li R. (2014). Noninvasive prenatal diagnosis of common aneuploidies by semiconductor sequencing. *Proc. Natl. Acad. Sci. U S A.* 111 7415–7420. 10.1073/pnas.1321997111 24799683 PMC4034209

[B22] Livergood M. C. LeChien K. A. Trudell A. S. (2017). Obesity and cell-free DNA “no calls”: is there an optimal gestational age at time of sampling? *Am. J. Obstet. Gynecol.* 216 .e411–.e413. 10.1016/j.ajog.2017.01.011 28153663

[B23] Lo Y. M. Corbetta N. Chamberlain P. F. Rai V. Sargent I. L. Redman C. W. (1997). Presence of fetal DNA in maternal plasma and serum. *Lancet (London, England)* 350 485–487. 10.1016/S0140-6736(97)02174-0 9274585

[B24] Noh J. J. Ryu H. M. Oh S. Y. Choi S. J. Roh C. R. Kim J. H. (2019). A two-year experience of non-invasive prenatal testing (NIPT) at an urban tertiary medical center in South Korea. *Taiwanese J. Obstet. Gynecol.* 58 545–551. 10.1016/j.tjog.2019.05.021 31307749

[B25] National Society of Genetic Counselors (NSGC) (2016). *Prenatal Cell-Free DNA Screening.* Available online at: https://www.nsgc.org/p/bl/et/blogaid=805

[B26] Palomaki G. E. Kloza E. M. Lambert-Messerlian G. M. Haddow J. E. Neveux L. M. Ehrich M. (2011). DNA sequencing of maternal plasma to detect Down syndrome: an international clinical validation study. *Genet. Med.* 13 913–920. 10.1097/GIM.0b013e3182368a0e 22005709

[B27] Pös O. Budiš J. Szemes T. (2019). Recent trends in prenatal genetic screening and testing. *F1000Research* 8:F1000FacultyRev-764. 10.12688/f1000research.16837.1 31214330 PMC6545823

[B28] Rather R. A. Dhawan V. Saha S. C. (2019). Non-invasive prenatal rhesus D genotyping using cell-free foetal DNA. *Ind. J. Med. Res.* 150 62–66. 10.4103/ijmr.IJMR_1787_17 PMC6798610 31571630

[B29] Rose N. C. Kaimal A. J. Dugoff L. Norton M. E. (2020). Screening for fetal chromosomal abnormalities: ACOG practice bulletin, number 226. *Obstet. Gynecol.* 136 e48–e69. 10.1097/AOG.0000000000004084 32804883

[B30] Sehnert A. J. Rhees B. Comstock D. de Feo E. Heilek G. Burke J. Rava R. P. (2011). Optimal detection of fetal chromosomal abnormalities by massively parallel DNA sequencing of cell-free fetal DNA from maternal blood. *Clin. Chem.* 57 1042–1049. 10.1373/clinchem.2011.165910 21519036

[B31] Sparks A. B. Struble C. A. Wang E. T. Song K. Oliphant A. (2012a). Noninvasive prenatal detection and selective analysis of cell-free DNA obtained from maternal blood: evaluation for trisomy 21 and trisomy 18. *Am. J. Obstet. Gynecol.* 206:319.e1-9. 10.1016/j.ajog.2012.01.030 22464072

[B32] Sparks A. B. Struble C. A. Wang E. T. Song K. Oliphant A. (2012b). Noninvasive prenatal detection and selective analysis of cell-free DNA obtained from maternal blood: evaluation for trisomy 21 and trisomy 18. *Am. J. Obstet. Gynecol.* 206:319.e1-9. 10.1016/j.ajog.2012.01.030 22464072

[B33] Sparks A. B. Wang E. T. Struble C. A. Barrett W. Stokowski R. McBride C. (2012c). Selective analysis of cell-free DNA in maternal blood for evaluation of fetal trisomy. *Prenatal Diag.* 32 3–9. 10.1002/pd.2922 22223233 PMC3500507

[B34] Srinivasan A. Bianchi D. W. Huang H. Sehnert A. J. Rava R. P. (2013). Noninvasive detection of fetal subchromosome abnormalities via deep sequencing of maternal plasma. *Am. J. Hum. Genet.* 92 167–176. 10.1016/j.ajhg.2012.12.006 23313373 PMC3567270

[B35] Wapner R. J. Lewis D. (2002). Genetics and metabolic causes of stillbirth. *Sem. Perinatol.* 26 70–74. 10.1053/sper.2002.29853 11876569

[B36] Zimmermann B. Hill M. Gemelos G. Demko Z. Banjevic M. Baner J. (2012). Non-invasive prenatal aneuploidy testing at chromosomes 13, 18, 21, X, and Y, using targeted sequencing of polymorphic loci. *Prenatal Diag.* 32 1233–1241. 10.1002/pd.3993 23108718 PMC3548605

